# High occurrence of β-lactamase-producing *Salmonella* Heidelberg from poultry origin

**DOI:** 10.1371/journal.pone.0230676

**Published:** 2020-03-31

**Authors:** Andrei I. S. Souza, Mauro M. S. Saraiva, Monique R. T. Casas, Gustavo M. Oliveira, Marita V. Cardozo, Valdinete P. Benevides, Fernanda O. Barbosa, Oliveiro C. Freitas Neto, Adriana M. Almeida, Angelo Berchieri

**Affiliations:** 1 Department of Veterinary Pathology, Laboratory of Avian Pathology, School of Agricultural and Veterinarian Sciences, São Paulo State University (Unesp), Jaboticabal, São Paulo, Brazil; 2 Agricultural and Livestock Microbiology Postgraduation Program, School of Agricultural and Veterinarian Sciences, São Paulo State University (Unesp), Jaboticabal, São Paulo, Brazil; 3 Nucleus of Enteric Diseases and Infections by Special Pathogens of the Center for Bacteriology of the Adolfo Lutz Institute, São Paulo, São Paulo, Brazil; 4 Department of Veterinary Pathology, Laboratory of Microbiology, School of Agricultural and Veterinarian Sciences, São Paulo State University (Unesp), Jaboticabal, São Paulo, Brazil; 5 Veterinary Medicine Postgraduation Program, School of Agricultural ad Veterinarian Sciences, São Paulo State University (Unesp), Jaboticabal, São Paulo, Brazil; 6 Federal University of Minas Gerais (UFMG), Belo Horizonte, Minas Gerais, Brazil; USDA-Agricultural Research Service, UNITED STATES

## Abstract

*Salmonella* Heidelberg is commonly reported in foodborne outbreaks around the world, and chickens and poultry products are known as important source of these pathogen. Multidrug-resistant *S*. Heidelberg strains are disseminated into poultry production chair, which can lead to severe clinical infections in humans and of difficult to treat. This study aimed at evaluating the β-lactam susceptibility and genotypic relatedness of *Salmonella* Heidelberg at Brazilian poultry production chain. Sixty-two *S*. Heidelberg strains from poultry production chain (poultry, poultry meat and poultry farm) were used. All strains were evaluated to antimicrobial susceptibility by diffusion disk test, as well as β-lactam resistance genes. Genotypic relatedness was assessed by Pulsed-Field Gel Eletrophoresis, using *Xba1* restriction enzyme. Forty-one strains were characterized as multidrug-resistant according to phenotype characterization. The resistance susceptibility revealed 31 distinct profiles, with higher prevalence of streptomycin (61/62), nalidixic acid (50/62), tetracycline (43/62) and β-lactam drugs (37/62). *bla*_CMY-2_ was the more frequent β-lactamase gene found (38/62); other resistance genes found were *bla*_CTX-M_ (2/62), *bla*_SHV_ (3/62) and *bla*_TEM-1_ (38/62). No carbapenemase genes was found. The Pulsed-Field Gel Electrophoresis showed 58 different profiles. Strains with a larger number of antimicrobial resistance were grouped into ten major clusters apart from others. The spread of resistance by *ampC* continues to rise, thereby turning concern to public health, since the β-lactam antimicrobials are used as a therapeutic treatment in humans.

## Introduction

The non-typhoid *Salmonella* (NTS) serovar Heidelberg (SH) is frequently found affecting humans and animals [1–5]. This pathogen has been commonly isolated in food-borne outbreaks from humans through consumption of poultry and pork-derived products, as well as dairy products [6]. SH is a pathogen of nonspecific host characterized by has a variety of infection sources and easy bacterial dissemination, due to their antigenic composition [7].

In the last years, SH has been reported causing outbreaks at 13 USA states, which 33 hospitalizations [2], and confirmed as the most frequent serovar involved in human diseases (21.6%) linked to poultry meat consumption (49,9%) [5]. Moreover, the high prevalence of multidrug-resistant (MDR) SH has been identified, including third generation cephalosporins [8–10], critical importance drugs to public health.

The antimicrobial use in animal production is a common practice, but it has a different procedure from different parts of the world. In the United States of America and European Union, the use of antimicrobials is limited to veterinarian prescription, and they should not be used to animal performance purpose. On the other hand, in Brazil some antibiotic groups are allowed to be used in animal production system to treat or to prevent infections or even as growth promoters [11,12]. Veterinary prescription is also required but sometimes failures in the official surveillance of antimicrobial use can lead to misuse. It is known that off-label use of some antimicrobials make a selective pressure which have been associated with quickly increased of bacterial resistance in Enterobacteriaceae species, as *E*. *coli*, from farms animals [13,14].

Currently, the class of β-lactam antimicrobials have been widely used to treat serious infections in humans and animals, including third and fourth generations of cephalosporins [15,16]. However, the bacterial resistance to cephalosporins has been found in *Salmonella* serovars, including Heidelberg from humans [4], poultry [9,17], and poultry meat [5], all of them presenting a diverse MDR pattern. Moreover, strains of MDR SH have been reported in the USA in outbreaks caused by chicken meat [10].

In Enterobacteriaceae species, the enzymatic inhibition is the main β-lactam resistance mechanism found. Both, Extended Spectrum β-Lactamase (ESBL) and Restrict Spectrum β-Lactamase (AmpC) are most common enzymes synthetized by *Salmonella* spp. [18–21], as well as most frequently found in Enterobacteriaceae isolated from poultry meat [22].

In this scenario, wherein ESBL/AmpC-producing bacteria is not only limited to hospitals and healthcare system but reaches food animals and food chain production [22,23], the spread of resistant *Salmonella* Heidelberg is a relevant public health issue. Furthermore, in view of the recent concern in the field with the frequent appearance of the this serovar resistant to different antimicrobials, this work aimed to evaluate the β-lactam susceptibility and genotypic relatedness of *Salmonella* Heidelberg, to provide information on the Brazilian scenario.

## Materials and methods

### *Salmonella* Heidelberg isolates

Sixty-two SH isolates were used: 20 from the Avian Pathology Laboratory (FCAV, Unesp Jaboticabal, São Paulo, BR) database and 42 from Adolfo Lutz Institute (IAL, São Paulo, BR) database (S1 Table). All SH isolates were obtained from poultry-relatedness samples, and categorized into three types: Poultry (sampled from cloacal swabs and cecal contents); Poultry farm (sampled from drag swabs, poultry feeders and drinkers); Poultry meat (sampled from product ready to consumption, in nature or processed). All strains were submitted to serovar confirmation by molecular assay using specific primers [24] (S1 File).

### Antimicrobial susceptibility testing

All 62 SH strains were submitted to the antimicrobial susceptibility using the disk diffusion test [25] and breakpoints used according to the recommendations of the Clinical and Laboratory Standards Institute [26]. The antimicrobials used are shown in S2 Table. Strains which presented resistance to three or more antimicrobial drug class used were considered MDR.

### β-lactam resistance genes

#### DNA extraction

All 62 SH strains were subjected to DNA extraction using the PureLink ^™^ Genomic DNA Kit (K182001, Invitrogen—Thermo Fisher Scientific, USA) following manufacturer's recommendations. The extracted DNA quantity was evaluated by spectrophotometer, DeNovix DS-11 + (DeNovix Inc., Delaware, USA), in nanogram per microliter (ng/μL).

#### Resistance genes

The presence of β-lactam resistance genes was evaluated by polymerase chain reaction (PCR) using specific primers [27] (S3 Table). The master mix concentrations and amplification conditions used in this study are described in S4 and S5 Tables.

The fragments were visualized from 5 μL of the amplicons along with 1 μL of Loading Dye buffer (Invitrogen, Thermo Fisher Scientific, USA) in the 1.5% agarose gel (Sigma Aldrich, Missouri, USA) stained with SyBr Safe DNA Gel Stain (Invitrogen, Thermo Fisher Scientific, USA). It was submitted to electrophoretic run under the 4V / cm conditions of the well (Bio-Rad Laboratories, USA) for 45 minutes. Then, the gel was subjected to UV light in a Gel Doc EZ Gel Documentation System (Bio-Rad Laboratories, USA).

### Pulsed-Field Gel Electrophoresis (PFGE)

PFGE was performed using *Xba*I (Sigma Aldrich, Missouri, USA) protocol [28]. Then, dendrogram was constructed using the Bionumerics version 7.1 (Applied Maths, Sint-Martens-Latem, Belgium) applying the Unweighted Pair Group Method with Arithmetic Mean method using the Dice coefficient with 1% tolerance and 0.5% of optimization.

## Results and discussion

### Antimicrobial susceptibility testing

According to the phenotypic susceptibility test, the prevalence of resistance was observed for streptomycin (S; 98.3%), nalidixic acid (NAL; 80.6%), tetracycline (T; 69.3%), cefotaxime (CTX; 59.7%), ampicillin (AMP; 58.1%) amoxicillin (AMX; 58.1%), cefoxitin (FOX; 56.4%), amoxicillin-clavulanate (AMC; 56,4%) and ceftiofur (CEF; 54.8%). In contrast, the lowest prevalence of resistance was observed for chloramphenicol (C; 1.6%) and imipenem (IMP; 4.8%). No resistance was observed against norfloxacin (NOR), amikacin (AK), gentamicin (GM) and trimethoprim-sulfamethoxazole (SXT) (Table 1).

**Table 1:** Antimicrobial resistance frequency in phenotypic test of 62 *Salmonella* Heidelberg strains collected from poultry, poultry meat and poultry farms.

Antimicrobials	Frequency of resistance			
	Poultry	Poultry meat	Poultry farms	Total (%)
Ciprofloxacin	0/10	0/20	1/32	1.6% (1/62)
Nalidixic acid	9/10	20/20	21/32	80.6% (50/62)
Enrofloxacin	2/10	4/20	12/32	29.1% (18/62)
Norfloxacin	0/10	0/20	0/32	0
Amikacin	0/10	0/20	0/32	0
Kanamycin	1/10	0/20	7/32	12.9% (8/62)
Streptomycin	10/10	19/20	32/32	98.3% (61/62)
Gentamicin	0/10	0/20	0/32	0
Ampicilin	8/10	17/20	11/32	58.1% (36/62)
Amoxilin	8/10	17/20	11/32	58.1% (36/62)
Imipenem	0/10	0/20	3/32	4.8% (3/62)
Ceftiofur	8/10	15/20	11/32	54.8% (34/62)
Cefotaxime	8/10	17/20	12/32	59.7% (37/62)
Cefoxitin	6/10	17/20	12/32	56.4% (35/62)
Amoxilin-clavulanate	8/10	17/20	10/32	56.4% (35/62)
Nitrofurantoin	10/10	9/20	8/32	43.5% (27/62)
Chloramphenicol	-	-	1/32	1.6% (1/62)
Tetracycline	9/10	20/20	14/32	69.3% (43/62)
Sulfamethoxazole-trimethoprim	0/10	0/20	0/32	0

Quinolones and fluoroquinolones [Ciprofloxacin, Nalidixic acid, Enrofloxacin, Norfloxacin]; Aminoglycosides [Amikacin, Kanamycin, Streptomycin, Gentamicin]; β-lactam [Penicillins (Ampicilin, Amoxilin), Carbapenems (Imipenem), Cephalosporins (Ceftiofur, Cefotaxime, Cefoxitin)]; β-lactam / β-lactamase inhibitor combinations [Amoxilin-clavulanate]; Nitrofurans [Nitrofurantoin]; Phenicols [Chloramphenicol]; Tetracyclines [Tetracycline]; Sulfonamide / Folate pathway inhinitors [Sulfamethoxazole-trimethoprim]

Based on antimicrobial susceptibility test, 41/62 (66,2%) of SH strains were resistant to three or more drug class and identified as MDR; nineteen (30.6%) of them resistant to five antimicrobial classes of the seven used in this work. These results reveled 31 different resistant profile from all SH, withal the most frequent pattern identified were NalFoxCefCtxAmcTNitAmxAmpS (12.1%) and NalFoxCefCtxAmcTAmxAmpS (11.3%) (S1 Table).

In this study, *Salmonella* Heidelberg showed resistance to some antimicrobials, as C, Nit and T, that are prohibited in Brazil on animal production since early 2000s [29,30]. However, the tetracycline is a commonly antimicrobial used in animals of production. Previous studies have been reported resistance to T after it was used as a growth promoter or performance enhancers in food animals [11,31]. The selective pressure by antimicrobial presence on environment favors the exponential resistance spread on gut, oral cavity and feces [32].

It is noteworthy that the prevalence of resistance among the 62 SH in this study was highest to NAL, S and β-lactams class drugs, including cephalosporin group ones. These results lead to a public health concern, since extended-spectrum cephalosporin (ESC) is indicated as first-line antibiotics for the gastrointestinal infections treatment caused by *Salmonella* spp. in humans [33].

In the present work, we found high occurrence of strains resistant to all β-lactam subclass used, including those combined to β-lactamase inhibitor (AMC). Penicillin associated to adjuvants has been used as alternative to β-lactam resistant bacteria in human infections caused both Gram-positive and Gram-negative species [15]. Furthermore, the drugs inhibitors, as clavulanic acid, acts under penicillinases and cephalosporinases, being used to distinguish ESBL-producing from AmpC-producing isolates [32,34]. In our results, all FOX resistant samples also presented resistance to AMC, suggesting that they are AmpC-producing SH and then confirmed by PCR.

Over to fifty percent of studied SH shown cephalosporin-resistance. These results are in accordance with previous works which reported cephalosporin-resistance in *Salmonella* from food-producing animals and poultry meat, since early 2000s [9,35,36]. Third-generation cephalosporin, as ceftiofur, have been frequently used in day-old chicks together with Marek’s vaccine [16], and it is related to short-term antimicrobial resistance in Enterobacteriaceae [13,14].

Despite that CEF is not a common cephalosporin used in human medicine, previous studies have shown a strict relationship between it and CTX resistance in Enterobacteriaceae [13,37]. We found complete agreement in that CEF and CTX resistance. This result suggests that ceftiofur could be used to access cefotaxime resistance by study model in poultry origin bacteria.

### β-lactam resistance genes

The frequency of ESBL, *ampC* and Carbapenemase genes in *Salmonella* Heidelberg from poultry origins are shown in Table 2. Extended spectrum β-lactamase genes were identified in seven of 62 SH strains studied: *bla*_CTX-M_, 3.22% (2/62); *bla*_SHV_, 4.83% (3/62); *bla*_TEM-1_, 3.22% (2/62). ESBL-producing Enterobacteriaceae harboring these genes are frequently found from animal and human origin [5,13,38,39]. None of other studied ESBL genes–*bla*_PSE_ and *bla*_OXA-2_ –were found in this study. The low prevalence of these determinants has been reported from animal hosts bacteria, whereas from humans, these isolates are often found [19,40].

**Table 2:** Resistance genes frequency of 62 *Salmonella* Heidelberg strains collected from poultry, poultry meat and poultry farms.

Gene		Poultry	Poultry meat	Poultry farms	Total (%)
**ESBL**	*bla*_CTX-M_	0/10	1/20	1/32	2 (3.22)
	*bla*_SHV_	0/10	1/20	2/32	3 (4.83)
	*bla*_TEM-1_	1/10	0/20	1/32	2 (3.22)
**AmpC**	*bla*_CMY-2_	8/10	17/20	13/32	38 (62.3)
**Carbapenemase**	*bla*_NDM_	0/10	0/20	0/32	0
	*bla*_OXA-48_	0/10	0/20	0/32	0

Differently from ESBL determinants, the *bla*_CMY-2_ gene was found in 62.3% (38/62) of SH strains (Table 2). The CMY-2 is related to resistance with second generation cephalosporins and penicillins through the production of β-lactamase AmpC [41] and it has been reported as major determinant of CEF resistance in Enterobacteriaceae from poultry source [42–44]. No others plasmid-mediated *ampC* genes investigated in our study were found in SH strains, including *bla*_FOX_ and *bla*_CMY-1_.

Usually, AmpC β-lactamase can occur as both a plasmid-mediated gene and hyperproduction of chromosomal *ampC*. The last mechanisms has instead of have been reported as the major one from animal origin bacteria [45,46], and it be related to mutations in enzyme regulatory [45]. Although chromosomal AmpC overproduction is the common resistance mechanism in AmpC-producing bacteria, in our results all CEF resistant SH shown plasmid-mediated cephamycinase. The increase of plasmid-mediated *bla*_CMY-2_ is associated with the CEF use in animal treatment infections, leading a new selective pressure to bacteria [5,16,35,47,48].

In the present study 36 strains displayed resistant to AMC, all of them presenting *bla*_CMY-2_ gene. The β-lactamase inhibitors use associated with β-lactams has confirmed the resistance caused by AmpC production, since the clavulanate acid (inhibitor) acts under the ESBL-producing strains [15] however, not under the AmpC-producing strains.

No carbapenemase coding genes were found, despite three different SH presented resistance against IMP (Table 2). The *bla*_NDM_ and *bla*_OXA-48_ gene-determinants, tested in this study, have been found in *Salmonella enterica* from different regions of the world [49–52]. Though no gene was found, we assume that IMP resistance in SH strains studied here, could harbor other carbapenemase group like those belonging the class A, usually found in others Enterobacteriaceae (e.g. *Klebsiella pneumoniae* and *Escherichia coli*) [53].

### Genotypic relatedness by PFGE

**Fig 1:**
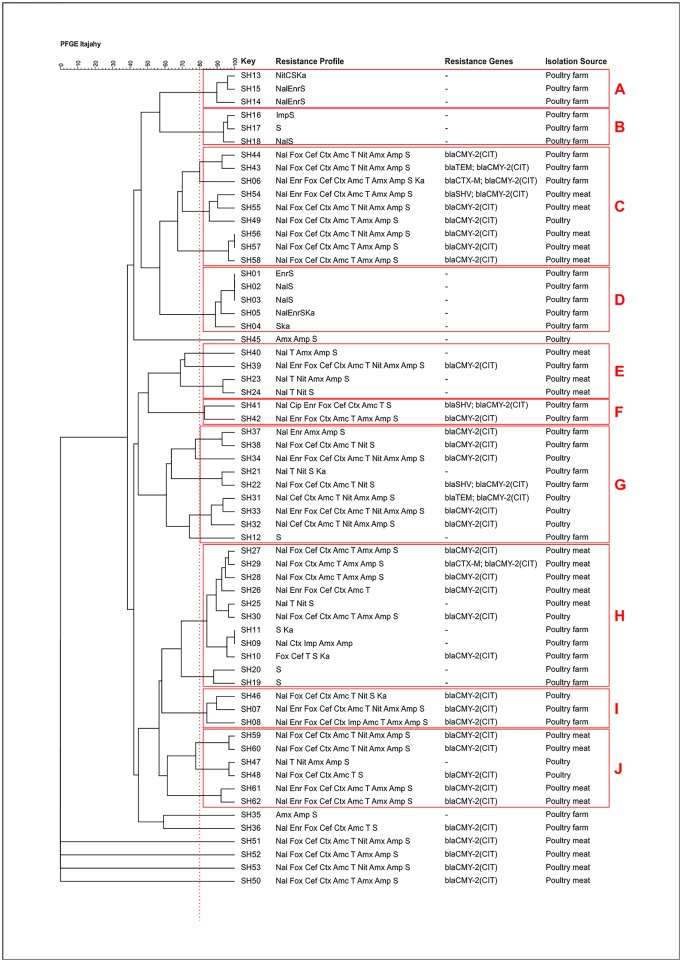
PFGE dendrogram of *Salmonella* Heidelberg isolates collected from poultry, poultry meat and poultry farms.

According to PFGE analysis carried from restriction using *XbaI*, studied SH strains were clustered in ten major groups as shown in Fig 1. Fifty-eight PFGE patterns were found among 62 *Salmonella* Heidelberg indicating a large diversity of these serovar from poultry sources. Studies in the USA have shown large diversity between *Salmonella enterica* serovar Heidelberg from food-producing animals [54]. Despite ours results shown high heterogeneity between isolates from different poultry sources (poultry, poultry meat and poultry farm), study with Heidelberg serovar from turkey-associated sources reveled extensive similarity between those isolates [55].

Compering PFGE with antimicrobial resistance results (phenotype and genotype ones), no relatedness between PFGE and resistance profiles. We assumed that these findings were due genetic diversity among SH isolates from poultry sources. Similarly, Lynne et al found large genetic diversity work with this serovar from cattle and swine sources [54].

## Conclusions

Our results showed high resistance characterized by both phenotypes and genotypes being related to *AmpC*. Strains resistant to a larger number of antimicrobials were also grouped into ten major clusters by PFGE. The present study reinforces that *Salmonella* Heidelberg from poultry origin is a serious hazard to public health that can act as foodborne pathogen. Therefore, measures to reduce antimicrobial resistance and to control *Salmonella* Heidelberg should be seriously addressed by the Brazilian poultry sector.

## Supporting information

S1 Table Information of the *Salmonella* Heidelberg strains used, including the antimicrobial susceptibility profile. (DOC)

S2 Table Class, concentrations and abbreviations of each antimicrobial drug used to disk diffusion test in this study. (DOCX)

S3 Table Primers sequences to evaluate the presence or absence of the β-lactamase resistance gene. (DOC)

S4 Table PCR master mix of β-lactam resistance genes. (DOC)

S5 Table Amplification conditions of β-lactam resistance genes. (DOC)

S1 File Master mix and PCR conditions to SH identification. (DOC)
