# Supplementary material for: High occurrence of β-lactamase-producing Salmonella Heidelberg from poultry origin
Source: PLoS One. 2020 Mar 31;15(3):e0230676. doi: 10.1371/journal.pone.0230676 (PMC7108700; doi:10.1371/journal.pone.0230676)
Supplement: S1 Table — (DOC) [file pone.0230676.s001.doc]

**S1 Table. Information of the *Salmonella* Heidelberg strains used, as well as the antimicrobial susceptibility profile.**

| **Strain** | **Year** | **Location*** | **Isolation Source** | **Phenotypic resistance** | **Resistance Gene** | **MDR^#^** |
| --- | --- | --- | --- | --- | --- | --- |
| SH 01 | 2002 | *-* | Poultry farm | Enr S | - | - |
| SH 02 | 2003 | *-* | Poultry farm | Nal S | - | - |
| SH 03 | 2004 | *-* | Poultry farm | Nal S | - | - |
| SH 04 | 2006 | *-* | Poultry farm | S Ka | - | - |
| SH 05 | 2010 | PR | Poultry farm | Nal Enr S Ka | - | - |
| SH 06 | 2012 | PR | Poultry farm | Nal Enr Fox Cef Ctx Amc T Amx Amp S Ka | *bla*_CTX-M_; *bla*_CMY-2(CIT)_ | MDR |
| SH 07 | 2014 | PR | Poultry farm | Nal Enr Fox Cef Ctx Amc T Nit Amx Amp S | *bla*_CMY-2(CIT)_ | MDR |
| SH 08 | 2015 | SC | Poultry farm | Nal Enr Fox Cef Ctx Imp Amc T Amx Amp S | *bla*_CMY-2(CIT)_ | MDR |
| SH 09 | 2008 | *-* | Poultry farm | Nal Ctx Imp Amx Amp | *-* | MDR |
| SH 10 | 2008 | *-* | Poultry farm | Fox Cef T S Ka | *bla*_CMY-2(CIT)_ | MDR |
| SH 11 | 2005 | *-* | Poultry farm | S Ka | - | - |
| SH 12 | 1983 | *-* | Poultry farm | S | - | - |
| SH 13 | 1984 | *-* | Poultry farm | Nit C S | - | MDR |
| SH 14 | 2000 | *-* | Poultry farm | Nal Enr S Ka | - | - |
| SH 15 | 2003 | *-* | Poultry farm | Nal Enr S | - | - |
| SH 16 | 2004 | *-* | Poultry farm | Imp S | - | - |
| SH 17 | 2004 | *-* | Poultry farm | S | - | - |
| SH 18 | 2005 | *-* | Poultry farm | Nal S | - | - |
| SH 19 | 2005 | *-* | Poultry farm | S | - | - |
| SH 20 | 2006 | *-* | Poultry farm | S | - | - |
| SH 21 | 2012 | Londrina/PR | Poultry farm | Nal T Nit S Ka Pe | - | - |
| SH 22 | 2012 | Cascavel/PR | Poultry farm | Nal Fox Cef Ctx Amc T Nit S | *bla*_SHV_; *bla*_CMY-2(CIT)_ | MDR |
| **S1 Table (continued). Information of the *Salmonella* Heidelberg strains used, as well as the antimicrobial susceptibility profile.** | | | | | | |
| SH 23 | 2014 | São Paulo/SP | Poultry meat | Nal T Nit Amx Amp S | - | - |
| SH 24 | 2014 | São Paulo/SP | Poultry meat | Nal T Nit S | - | - |
| SH 25 | 2014 | São Paulo/SP | Poultry meat | Nal T Nit S | - | - |
| SH 26 | 2014 | São Paulo/SP | Poultry meat | Nal Enr Fox Cef Ctx Amc T | *bla*_CMY-2(CIT)_ | MDR |
| SH 27 | 2014 | São Paulo/SP | Poultry meat | Nal Fox Cef Ctx Amc T Amx Amp S | *bla*_CMY-2(CIT)_ | MDR |
| SH 28 | 2014 | São Paulo/SP | Poultry meat | Nal Fox Ctx Amc T Amx Amp S | *bla*_CMY-2(CIT)_ | MDR |
| SH 29 | 2014 | São Paulo/SP | Poultry meat | Nal Fox Ctx Amc T Amx Amp S | *bla*_CTX-M_; *bla*_CMY-2(CIT)_ | MDR |
| SH 30 | 2014 | São Paulo/SP | Poultry | Nal Fox Cef Ctx Amc T Amx Amp S | *bla*_CMY-2(CIT)_ | MDR |
| SH 31 | 2014 | São Paulo/SP | Poultry | Nal Cef Ctx Amc T Nit Amx Amp S | *bla*_TEM_; *bla*_CMY-2(CIT)_ | MDR |
| SH 32 | 2014 | São Paulo/SP | Poultry | Nal Cef Ctx Amc T Nit Amx Amp S | *bla*_CMY-2(CIT)_ | MDR |
| SH 33 | 2014 | São Paulo/SP | Poultry | Nal Enr Fox Cef Ctx Amc T Nit Amx Amp S | *bla*_CMY-2(CIT)_ | MDR |
| SH 34 | 2014 | São Paulo/SP | Poultry | Nal Enr Fox Cef Ctx Amc T Nit Amx Amp S | *bla*_CMY-2(CIT)_ | MDR |
| SH 35 | 2014 | Campinas/SP | Poultry farm | Amx Amp S | - | - |
| SH 36 | 2014 | Campinas/SP | Poultry farm | Nal Enr Fox Cef Ctx Amc T S | *bla*_CMY-2(CIT)_ | MDR |
| SH 37 | 2014 | Campinas/SP | Poultry farm | Nal Enr Amx Amp S | *bla*_CMY-2(CIT)_ | - |
| SH 38 | 2014 | Campinas/SP | Poultry farm | Nal Fox Cef Ctx Amc T Nit S | *bla*_CMY-2(CIT)_ | MDR |
| SH 39 | 2014 | Campinas/SP | Poultry farm | Nal Enr Fox Cef Ctx Amc T Nit Amx Amp S | *bla*_CMY-2(CIT)_ | MDR |
| SH 40 | 2015 | São Paulo/SP | Poultry meat | Nal T Amx Amp S | - | MDR |
| SH 41 | 2015 | Londrina/PR | Poultry farm | Nal Cip Enr Fox Cef Ctx Amc T S | *bla*_SHV_; *bla*_CMY-2(CIT)_ | MDR |
| SH 42 | 2015 | Uberlândia/MG | Poultry farm | Nal Enr Fox Ctx Amc T Amx Amp S | *bla*_CMY-2(CIT)_ | MDR |
| SH 43 | 2017 | São Paulo/SP | Poultry farm | Nal Fox Cef Ctx Amc T Nit Amx Amp S | *bla*_TEM_; *bla*_CMY-2(CIT)_ | MDR |
| SH 44 | 2017 | São Paulo/SP | Poultry farm | Nal Fox Cef Ctx Amc T Nit Amx Amp S | *bla*_CMY-2(CIT)_ | MDR |
| SH 45 | 2016 | Avaré/SP | Poultry | Amx Amp S | - | - |
| SH 46 | 2016 | Avaré/SP | Poultry | Nal Fox Cef Ctx Amc T Nit S Ka | *bla*_CMY-2(CIT)_ | MDR |
| **S1 Table (continued). Information of the *Salmonella* Heidelberg strains used, as well as the antimicrobial susceptibility profile.** | | | | | | |
| SH 47 | 2016 | Avaré/SP | Poultry | Nal T Nit Amx Amp S | - | MDR |
| SH 48 | 2016 | Avaré/SP | Poultry | Nal Fox Cef Ctx Amc T S | *bla*_CMY-2(CIT)_ | MDR |
| SH 49 | 2016 | Avaré/SP | Poultry | Nal Fox Cef Ctx Amc T Amx Amp S | *bla*_CMY-2(CIT)_ | MDR |
| SH 50 | 2018 | Avaré/SP | Poultry meat | Nal Fox Cef Ctx Amc T Amx Amp S | *bla*_CMY-2(CIT)_ | MDR |
| SH 51 | 2018 | Avaré/SP | Poultry meat | Nal Fox Cef Ctx Amc T Nit Amx Amp S | *bla*_CMY-2(CIT)_ | MDR |
| SH 52 | 2018 | Avaré/SP | Poultry meat | Nal Fox Cef Ctx Amc T Amx Amp S | *bla*_CMY-2(CIT)_ | MDR |
| SH 53 | 2018 | Avaré/SP | Poultry meat | Nal Fox Cef Ctx Amc T Nit Amx Amp S | *bla*_CMY-2(CIT)_ | MDR |
| SH 54 | 2018 | Avaré/SP | Poultry meat | Nal Enr Fox Cef Ctx Amc T Amx Amp S | *bla*_SHV_; *bla*_CMY-2(CIT)_ | MDR |
| SH 55 | 2018 | Avaré/SP | Poultry meat | Nal Fox Cef Ctx Amc T Nit Amx Amp S | *bla*_CMY-2(CIT)_ | MDR |
| SH 56 | 2018 | Avaré/SP | Poultry meat | Nal Fox Cef Ctx Amc T Nit Amx Amp S | *bla*_CMY-2(CIT)_ | MDR |
| SH 57 | 2018 | Avaré/SP | Poultry meat | Nal Fox Cef Ctx Amc T Amx Amp S | *bla*_CMY-2(CIT)_ | MDR |
| SH 58 | 2018 | Avaré/SP | Poultry meat | Nal Fox Cef Ctx Amc T Amx Amp S | *bla*_CMY-2(CIT)_ | MDR |
| SH 59 | 2018 | Avaré/SP | Poultry meat | Nal Fox Cef Ctx Amc T Nit Amx Amp S | *bla*_CMY-2(CIT)_ | MDR |
| SH 60 | 2018 | Avaré/SP | Poultry meat | Nal Fox Cef Ctx Amc T Nit Amx Amp S | *bla*_CMY-2(CIT)_ | MDR |
| SH 61 | 2018 | Avaré/SP | Poultry meat | Nal Enr Fox Cef Ctx Amc T Amx Amp S | *bla*_CMY-2(CIT)_ | MDR |
| SH 62 | 2018 | Avaré/SP | Poultry meat | Nal Enr Fox Cef Ctx Amc T Amx Amp S | *bla*_CMY-2(CIT)_ | MDR |

*all of them were isolated in Brazil. ^#^MDR characterization were based on phenotypic resistance results.

PR - Paraná; SC - Santa Catarina; SP - São Paulo; MG - Minas Gerais. Cip: Ciprofloxacin, Nal: Nalidixic acid; Enr: Enrofloxacin, Nor: Norfloxacin, Ak: Amikacin, Ka: Kanamycin, S: Streptomycin, Gm: Gentamicin, Amp: Ampicilin, Amx: Amoxilin, Imp: Imipenem, Cef: Ceftiofur, CTX: Cefotaxime, Fox: Cefoxitin, Amc: Amoxilin-clavulanate, Nit: Nitrofurantoin, C: Chloramphenicol, T: Tetracycline, SXT: Trimethoprim-sulfamethoxazole, MDR: Multidrug-resistance
