# Supplementary material for: High occurrence of β-lactamase-producing Salmonella Heidelberg from poultry origin
Source: PLoS One. 2020 Mar 31;15(3):e0230676. doi: 10.1371/journal.pone.0230676 (PMC7108700; doi:10.1371/journal.pone.0230676)
Supplement: S2 Table — (DOCX) [file pone.0230676.s002.docx]

**S2 Table.** **Class, concentrations and abbreviations of each Antimicrobial drug used to disk diffusion test in this study.**

| **Class** | **Antimicrobial** | **Concentration** | **Abbreviation** |
| --- | --- | --- | --- |
| Quinolones and fluoroquinolones | Ciprofloxacin | 5 µg | CIP |
|  | Nalidixic acid | 30 µg | NAL |
|  | Enrofloxacin | 5 µg | ENR |
|  | Norfloxacin | 10 µg | NOR |
| Aminoglycosides | Amikacin | 30 µg | AK |
|  | Kanamycin | 30 µg | KA |
|  | Streptomycin | 10 µg | S |
|  | Gentamicin | 10 µg | GM |
| β-lactam (Penicillins) | Ampicilin | 10 µg | AMP |
|  | Amoxilin | 10 µg | AMX |
| β-lactam (Carbapenems) | Imipenem | 10 µg | IMP |
| β-lactam (Cephalosporins) | Ceftiofur | 30 µg | CEF |
|  | Cefotaxime |  | CTX |
|  | Cefoxitin | 30 µg | FOX |
| β-lactam/ β-lactamase inhibitor combinations | Amoxilin-clavulanate | 20/10 µg | AMC |
| Nitrofurans | Nitrofurantoin | 300 µg | NIT |
| Phenicols | Chloramphenicol | 30 µg | C |
| Tetracyclines | Tetracycline | 30 µg | T |
| Folate pathway inhinitors | Trimethoprim-sulfamethoxazole | 1.25/23.75 µg | SXT |
