# Supplementary material for: High occurrence of β-lactamase-producing Salmonella Heidelberg from poultry origin
Source: PLoS One. 2020 Mar 31;15(3):e0230676. doi: 10.1371/journal.pone.0230676 (PMC7108700; doi:10.1371/journal.pone.0230676)
Supplement: S3 Table — (DOC) [file pone.0230676.s003.doc]

**S3 Table. Primers sequences to evaluate the presence or absence of the β-lactamase** resistance gene.

| **Resistance type** | **Gene** | **Sequence (5’ > 3’)** | **Size (bp)** | **Reference** |
| --- | --- | --- | --- | --- |
| ESBL | *bla*_TEM_ | GCACGAGTGGGTTACATCGA | 310 | [24] |
|  |  | GGTCCTCCGATCGTTGTCAG |  |  |
|  | *bla*_SHV_ | GGGTTATTCTTATTTGTCGC | 930 | [11] |
|  |  | TTAGCGTTGCCAGTGCTC |  |  |
|  | *bla*_CTX-M_ | SCSATGTGCAGYACCAGTAA | 554 | [11] |
|  |  | CCGCRAT ATGRTTGGTGGTG |  |  |
|  | *bla*_PSE-1_ | TTTGGTTCCGCGCTATCTG | 150 | [25] |
|  |  | TACTCCGAGCACCAAATCCG |  |  |
|  | *bla*_OXA-2_ | ATGGCAATCCGAATCTTCGC | 650 | [25] |
|  |  | TTATCGCGCAGCGTCCGAGT |  |  |
| ampC | *bla*_CMY_ (MOX) | GCTGCTCAAGGAGCACAGGAT | 520 | [24] |
|  |  | CACATTGACATAGGTGTGGTGC |  |  |
|  | *bla*_CMY-2_ (CIT) | TGGCAAGAACTGACAGGCAAA | 462 | [25] |
|  |  | TTTCTCCTGAACGTGGCTGGC |  |  |
|  | *bla*_FOX_ | AACATGGGGTATCAGGGAGATG | 190 | [26] |
|  |  | CAAAGCGCGTAACCGGATTGG |  |  |
| Carbapenemases | *bla*_OXA-48_ | GCGTGGTTAAGGATGAACAC | 438 | [27] |
|  |  | CATCAAGTTCAACCCAACCG |  |  |
|  | *bla*_NDM_ | GGTTTGGCGATCTGGTTTTC | 621 | [27] |
|  |  | CGGAATGGCTCATCACGATC |  |  |
