# Supplementary material for: High occurrence of β-lactamase-producing Salmonella Heidelberg from poultry origin
Source: PLoS One. 2020 Mar 31;15(3):e0230676. doi: 10.1371/journal.pone.0230676 (PMC7108700; doi:10.1371/journal.pone.0230676)
Supplement: S4 Table — (DOC) [file pone.0230676.s004.doc]

**S4 Table. PCR master mix of β**-lactam resistance genes.

**Reagents**

| **Resistance type** | ESBL | | | | | ampC | Carbapenamases |
| --- | --- | --- | --- | --- | --- | --- | --- |
| **Primers** | CTX-M | SHV | TEM-1 | PSE | OXA-2 | MOX*  FOX*  CIT^#^ | NDM  OXA-48 |
| ^1^Buffer | 1 X | 1 X | 1 X | 1 X | 1 X | 1 X | 1 X |
| ^1^MgCl_2_ | 3 mM | 3 mM | 3 mM | 3 mm | 1.5 mM | 1.5 mM | 1.5 mM |
| ^1^dNTP | 200 µM | 200 µM | 200 µM | 200 µM | 200 µM | 200 µM | 125 µM |
| ^1^Primer (each) | 1.2 pmol | 1.2 pmol | 1.2 pmol | 1.2 pmol | 1.2 pmol | 1.2 pmol*  0.8 pmol^#^ | 0.8 pmol |
| ^1^Taq DNA Pol | 1 U | 1 U | 1 U | 1 U | 1 U | 1.25 U | 1.5 U |
| DNA | 100 ng | 100 ng | 100 ng | 100 ng | 100 ng | 100 ng | 100 ng |
| **Final volume** | **25 µL** | **25 µL** | **25 µL** | **25 µL** | **25 µL** | **25 µL** | **25 µL** |

The Ultra-pure water^1^ was used to complete reaction volume. ^1^Sigma Aldrich, Missouri, USA;
