# Supplementary material for: High occurrence of β-lactamase-producing Salmonella Heidelberg from poultry origin
Source: PLoS One. 2020 Mar 31;15(3):e0230676. doi: 10.1371/journal.pone.0230676 (PMC7108700; doi:10.1371/journal.pone.0230676)
Supplement: S5 Table — (DOC) [file pone.0230676.s005.doc]

**S5 Table. Amplification conditions of β**-lactam resistance genes.

|  |  | **Primers** | | | | | | |
| --- | --- | --- | --- | --- | --- | --- | --- | --- |
| **Phase** | **Cycle**  **(repeat)** | CTX-M^a^ | SHV^b^ | MOX^c^  FOX^c^  CIT^c^ | TEM-1^b^ | PSE ^d^ | OXA-2^e^ | NDM^f^  OXA-48^f^ |
| Initial denaturation | 1 X | 94 ºC  180 s | 94 ºC  300 s | 94 ºC  180 s | 95 ºC  300 s | 95 ºC  420 s | 94 ºC  240 s | 94 ºC  600 s |
| Denaturation | 35 X^a^  30 X^b^  25 X^c^  40 X^d^  38 X^e^  36 X^f^ | 94 ºC  30 s | 94 ºC  60 s | 94 ºC  30 s | 95 ºC  60 s | 95 ºC  60 s | 94 ºC  30 s | 94 ºC  30 s |
| Annealing |  | 57.6 ºC  30 s | 55.5 ºC  60 s | 64 ºC  30 s | 55 ºC  60 s | 48 ºC  30 s | 55 ºC  30 s | 54 ºC  40 s |
| Extension |  | 72 ºC  45 s | 72 ºC  60 s | 72 ºC  60 s | 72 ºC  60 s | 72 ºC  30 s | 72 ºC  30 s | 72 ºC  40 s |
| Final Extension | 1 X | 72 ºC  300 s | 72 ºC  300 s | 72 ºC  420 s | 72 ºC  420 s | 72 ºC  420 s | 72 ºC  300 s | 72 ºC  300 s |
