# Supplementary material for: High occurrence of β-lactamase-producing Salmonella Heidelberg from poultry origin
Source: PLoS One. 2020 Mar 31;15(3):e0230676. doi: 10.1371/journal.pone.0230676 (PMC7108700; doi:10.1371/journal.pone.0230676)
Supplement: S1 File — (DOC) [file pone.0230676.s006.doc]

**S1 File.** **Master mix and PCR conditions to SH identification.**

The amplified gene region encodes an SH-specific methylase subunit type II restriction enzyme (GenBank accession number: ACF69659.1). Twenty microliters of the total reaction was composed of 4 μL of 5 x FIREPol Master Mix with 12.5 mM MgCl_2_ (Solis BioDyne, USA), 50 μM of the direct oligonucleotide primers [21] [SH-F (5'-TGT-TTGGAG CAT CAT CAG AA-3 ')] and reverse [SH-R (5'-GCT CAA CAT AAG GGA AGC AA-3')] (Invitrogen, Thermo Fisher Scientific, USA), 1 μl DNA and ultrapure water (Sigma Aldrich, USA) in sufficient quantity to (q.s.p.) complete the reaction.

The PCR conditions were carried out in MyCycler Thermal Cycler (Bio-Rad Laboratories, USA) with the following specifications: an initial cycle of denaturation at 95 ° C for 2 minutes; 30 cycles of denaturation at 95 ° C for 30 seconds, annealing at 60 ° C for 20 seconds and extension of the new DNA strand at 72 ° C for 20 seconds; and finally a single extension cycle at 72 for 5 minutes.

Then 5 μl of each amplified sample was diluted in 1 μl of 1: 500 Gel Red Nucleic Acid Stain (Biotium, California, USA) plus 1 μl of Loading Dye buffer (Fermentas, Thermo Fisher Scientific, USA). In addition, a negative control was used in each of the reactions that allowed the visualization of possible contaminants and inhibitors, replacing the genetic material with ultrapure water in the same quantity. In order to identify the molecular size of the generated amplicons, 5 μl of 100 base pair (bp) molecular marker (Fermentas, Thermo Fisher Scientific, USA) plus 2 μl of Gel Red Nucleic Acid Stain was used. Thus, electrophoresis was performed on 1.5% agarose gel (Sigma Aldrich, USA) under the conditions of 5 V / cm (Bio-Rad Laboratories, USA) for 1 hour. After the electrophoretic run, the gel was subjected to UV light on a photodocumentator Gel Doc EZ Gel Documentation System (Bio-Rad Laboratories, USA) for the visualization of the amplicons.
